# OP52 Green Breathing: What Is The Added Economic Value Of Carbon Minimal Medical Products? A Case Study Of Inhalers

**DOI:** 10.1017/S0266462325101104

**Published:** 2025-12-29

**Authors:** Sukanya Subramaniyan, Grace Hampson

## Abstract

**Introduction:**

Healthcare systems are substantial greenhouse gas emitters, responsible for five percent of emissions worldwide. Inhalers are particularly problematic, and while all inhalers have a negative impact, pressurized metered-dose inhalers (pMDIs) are the worst offenders. With carbon minimal pMDIs in development, this research evaluated the economic value that selected carbon minimal pMDIs could offer and explored how this value can be represented in economic evaluations.

**Methods:**

The analysis employed a partial economic evaluation framework. Given the carbon minimal and current standard pMDIs are expected to be clinically equivalent, clinical outcomes and downstream resources offset each other in an incremental analysis. Thus, the evaluation centered around reduction in carbon and incremental costs. Two approaches were undertaken: parallel and integrated evaluation. Environment impact (carbon footprint) was sourced from published life cycle assessments, dosage was based on international guidelines, and costs were taken from the British National Formulary. The cost of carbon used was the UK Treasury’s carbon value of GBP269 (USD370) per ton of carbon dioxide equivalent (CO2e).

**Results:**

Current selected pMDIs produce 103 kg to 150 kg of CO2e per person per year depending on which product is used, whereas the carbon minimal pMDIs produce 13 kg to 18 kg of CO2e per person per year, a reduction of around 88 percent. The undiscounted additional value of the selected carbon minimal pMDIs ranges from GBP776 (USD1,068) to GBP1,134 (USD1,561) per person with asthma over their lifetime. Based on these results, at a population level, if all people currently being prescribed pMDIs moved to a low carbon pMDIs, this could save carbon emissions to the value of GBP112 to 166 million (USD154 to 229 million) annually in the UK alone.

**Conclusions:**

The added value demonstrates the environmental and economic benefits available from switching from existing pMDIs to low carbon pMDIs where clinical outcomes are equivalent. This additional value can be reflected in economic evaluations as part of an HTA process, although challenges remain related to data availability and in the interpretation of environmental impact data in cases of non-dominance.

